# Legionnaires’ Disease Presenting With Erythema Multiforme in an Immunocompetent Patient

**DOI:** 10.7759/cureus.107654

**Published:** 2026-04-24

**Authors:** Ali Wayzani, Jony Dib, Josepha Bertrac

**Affiliations:** 1 Pulmonary Medicine, Hopital Alexandra Lepève, Centre Hospitalier de Dunkerque (CHD), Dunkerque, FRA; 2 Critical Care Medicine, Hopital Alexandra Lepève, Centre Hospitalier de Dunkerque (CHD), Dunkerque, FRA; 3 Critical Care Medicine, Hôpital Alexandra Lepève, Centre Hospitalier de Dunkerque (CHD), Dunkerque, FRA

**Keywords:** erythema multiforme-like lesions, extrapulmonary manifestation, legionella pneumophila, legionnaire's disease, skin lesions

## Abstract

Legionnaires’ disease is a severe form of pneumonia most commonly caused by Legionella pneumophila serogroup 1. While primarily affecting the lungs, extrapulmonary manifestations such as hyponatremia, renal dysfunction, and hepatic involvement are well described. In contrast, cutaneous manifestations are rare and remain poorly characterized.

We report the case of a 62-year-old immunocompetent man with a history of chronic alcohol use and active smoking who presented with fever, dyspnea, and productive cough. On admission, he was febrile (38.5°C), tachycardic, and had crackles over the right lower lung field. Laboratory evaluation revealed hyponatremia (132 mmol/L) and markedly elevated inflammatory markers (CRP 480 mg/L, procalcitonin 41 ng/mL). Chest imaging demonstrated right lower lobe pneumonia. Initial empiric therapy with amoxicillin-clavulanate was started.

Within 48 hours, the patient developed acute respiratory failure requiring intubation and ICU admission. Urinary antigen testing confirmed Legionella pneumophila infection, and antibiotic therapy was switched to levofloxacin and spiramycin. The course was complicated by severe ARDS (PaO₂/FiO₂ ratio 100) requiring prone positioning, and septic shock requiring vasopressors.

On day 9 of hospitalization (seven days after targeted therapy initiation), the patient developed a diffuse, non-pruritic erythematous maculopapular rash with targetoid lesions, involving the trunk and limbs, with palmar involvement and petechiae at the ankles, without mucosal involvement. A drug eruption was initially suspected; however, skin biopsy showed mild interkeratinocytic edema with superficial lymphocytic infiltrate and rare keratinocyte necrosis, without eosinophilia or features of toxidermia. Direct immunofluorescence and PCR testing for herpes simplex virus and Mycoplasma pneumoniae were negative. These findings supported a diagnosis of erythema multiforme (EM) secondary to Legionella infection.

The rash resolved spontaneously without specific treatment. The patient improved clinically, was extubated on day 12, weaned off vasopressors by day 10, and discharged on day 20 after completing antibiotic therapy.

This case illustrates a rare presentation of Legionnaires’ disease complicated by EM. Recognition of this association is important, as cutaneous findings may mimic drug reactions and lead to inappropriate modification of effective antimicrobial therapy. This report contributes to the limited literature describing dermatologic manifestations of Legionella infection and underscores the need for clinical awareness of this uncommon presentation.

## Introduction

Legionnaires’ disease is a severe form of community-acquired and nosocomial pneumonia caused by the intracellular Gram-negative bacillus Legionella, most commonly Legionella pneumophila serogroup 1. It is associated with significant morbidity and mortality, particularly in older adults and immunocompromised patients, and may require intensive care support. The organism is typically found in humid environments, including natural and man-made water systems such as cooling towers, spas, and plumbing networks, and is transmitted via inhalation of aerosolized contaminated water droplets [1].

Recent data from the European Centre for Disease Prevention and Control (ECDC) demonstrate a rising incidence of Legionnaires’ disease in Europe, with a notification rate of 2.4 cases per 100,000 population in 2021, the highest recorded since surveillance began. The disease predominantly affects males and individuals over 65 years of age [2].

Diagnosis is commonly established using urinary antigen testing for L. pneumophila serogroup 1, while culture on buffered charcoal yeast extract agar remains the gold standard [1].

Clinically, Legionnaires’ disease presents with fever, cough, dyspnea, and systemic features such as gastrointestinal symptoms and confusion. Extrapulmonary manifestations are uncommon, and cutaneous involvement is particularly rare and likely underreported. Reported skin findings are variable and may mimic drug-induced eruptions, creating diagnostic uncertainty [3].

Erythema multiforme (EM) is an acute, immune-mediated hypersensitivity reaction characterized by target lesions, most often triggered by infections such as herpes simplex virus or Mycoplasma pneumoniae. Its association with Legionella infection is rare but clinically relevant, as misdiagnosis as a drug reaction may lead to inappropriate discontinuation of essential antimicrobial therapy.

We report a case of Legionnaires’ disease associated with a cutaneous eruption consistent with EM, underscoring the importance of recognizing infection-related dermatologic manifestations in critically ill patients.

## Case presentation

A 62-year-old Caucasian man with a history of chronic alcohol consumption, active smoking, and prior shingles presented to the emergency department with a two-day history of fever, dyspnea, dizziness, and a productive cough. On admission, the patient was febrile, with a temperature of 38.5°C, tachycardic 102 BPM, with an O2 saturation of 94% on room air. Physical examination revealed coarse crackles in the right lower lung field without signs of respiratory distress. Neurological examination was unremarkable, and no skin lesions were noted.

**Figure 1:**
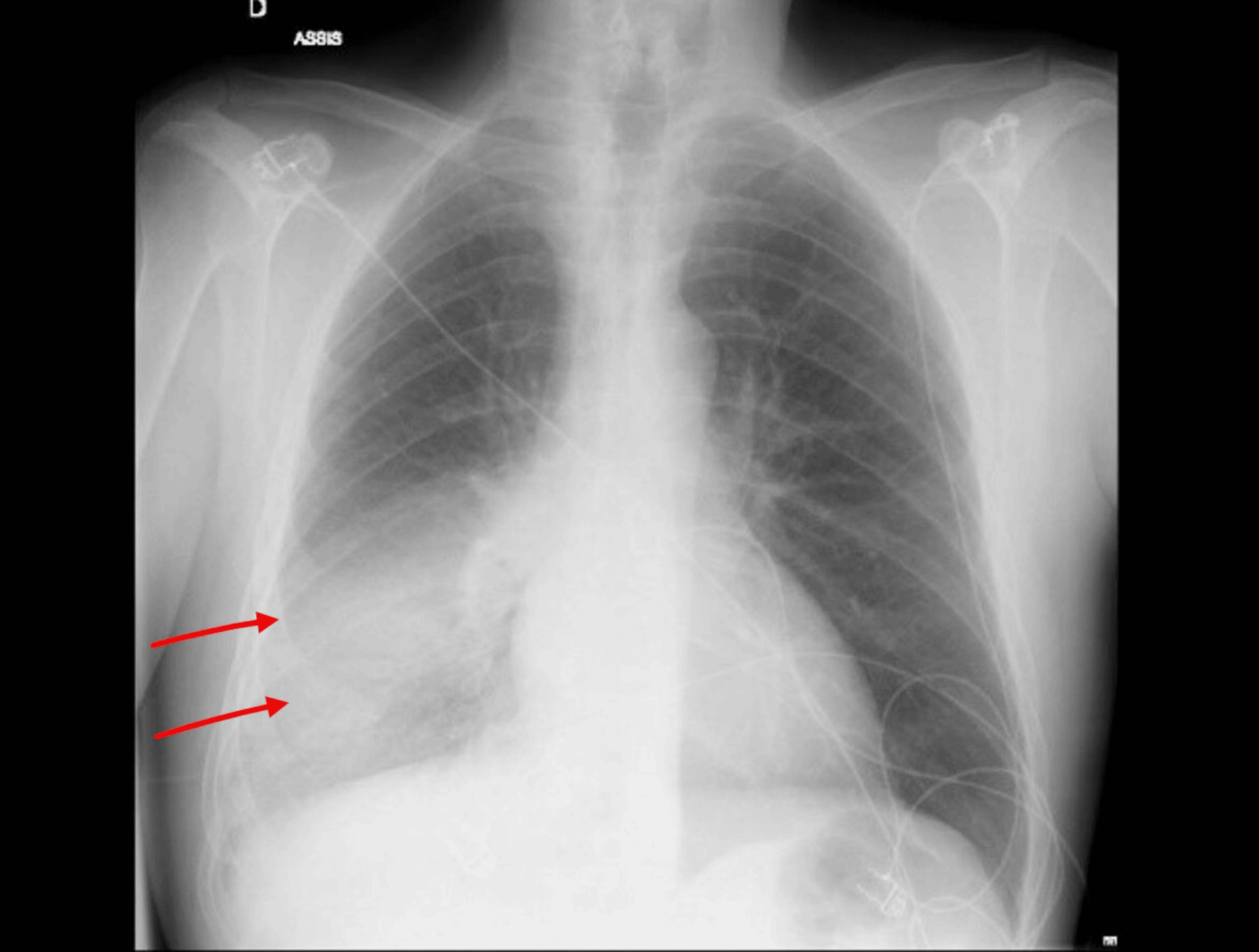
Right lower lung field consolidation

A chest radiograph demonstrated a right lower lobe consolidation consistent with lobar pneumonia (Figure 1).

Laboratory investigations taken after examination are presented in Table 1. We highlight the presence of hyponatremia with a sodium level of 132 mmol/L, and the marked elevation of Inflammatory markers with a C-reactive protein (CRP) of 480.6 mg/L, and a procalcitonin of 41.86 ng/ml.

**Table 1:** Labs results on admission

Test	Result	Units	Reference Values
Complete Blood Count with Differential			
Red blood cells	3.76	T/L	(4.50-5.80)
Hemoglobin	12.0	g/dL	(13.0-17.0)
Hematocrit	34.9	%	(39.0-51.0)
White blood count	10580	/mm3	(4000-10000)
Platelets	180000	/mm3	(150000-400000)
Neutrophils	96.6	%	-
Eosinophils	0.1	%	-
Basophils	0.4	%	-
Lymphocytes	2.0	%	-
Monocytes	0.9	%	-
Arterial blood gas			
Oxygen (L/min)	6		
Blood pH	7.34		(7.35-7.45)
Partial concentration of carbon dioxide	42.1	mmHg	(34.0-45.0)
Partial concentration of oxygen	37.8	mmHg	(72.0-103.0)
Bicarbonates	22.5	mmol/L	(22.2-28.3)
Base excess	-3.3		
Oxyhemoglobin	65.7	%	(90.0-95.0)
Saturation	67.2	%	(94.0-98.0)
Biochemistry			
Urea	0.44	g/L	(0.19-0.49)
Creatinine	6.9	mg/L	(6.0-11.0)
Sodium	132	mmol/L	(136-145)
Potassium	3.7	mmol/L	(3.4-4.5)
Chloride	101	mmol/L	(98-107)
Bicarbonates	20.5	mmol/L	(20.0-31.0)
Lactate	4.30	mmol/L	(0.50-2.20)
Bilirubin total	16.1	mg/L	(3.0-12.0)
Bilirubin direct	12.2	mg/L	(1.0-3.0)
Aspartate aminotransferase	133	U/L	(13-40)
Alanine aminotransferase	32	U/L	(7-40)
Gamma-glutamyl transferase	22	U/L	(<73)
Alkaline phosphatase	80	U/L	(46-116)
Lipase	81	U/L	(12-53)
CRP (C-Reactive protein)	480.6	mg/L	(<10.0)
Procalcitonin	41.86	ng/mL	(<0.05

Intravenous amoxicillin-clavulanate was initiated, and the patient was admitted to the infectious diseases ward. Two days later, the patient developed acute respiratory distress requiring endotracheal intubation and mechanical ventilation, and he was transferred to the intensive care unit. At admission, the CURB-65 score was estimated to be at least 1-2, suggesting a non-low-risk pneumonia. Lab tests showed severe hypoxemia with lactic acidosis and leukocytosis. Urine analysis and testing for legionella came back positive. Targeted therapy with intravenous levofloxacin and spiramycin was initiated as by local recommendations.

**Figure 2:**
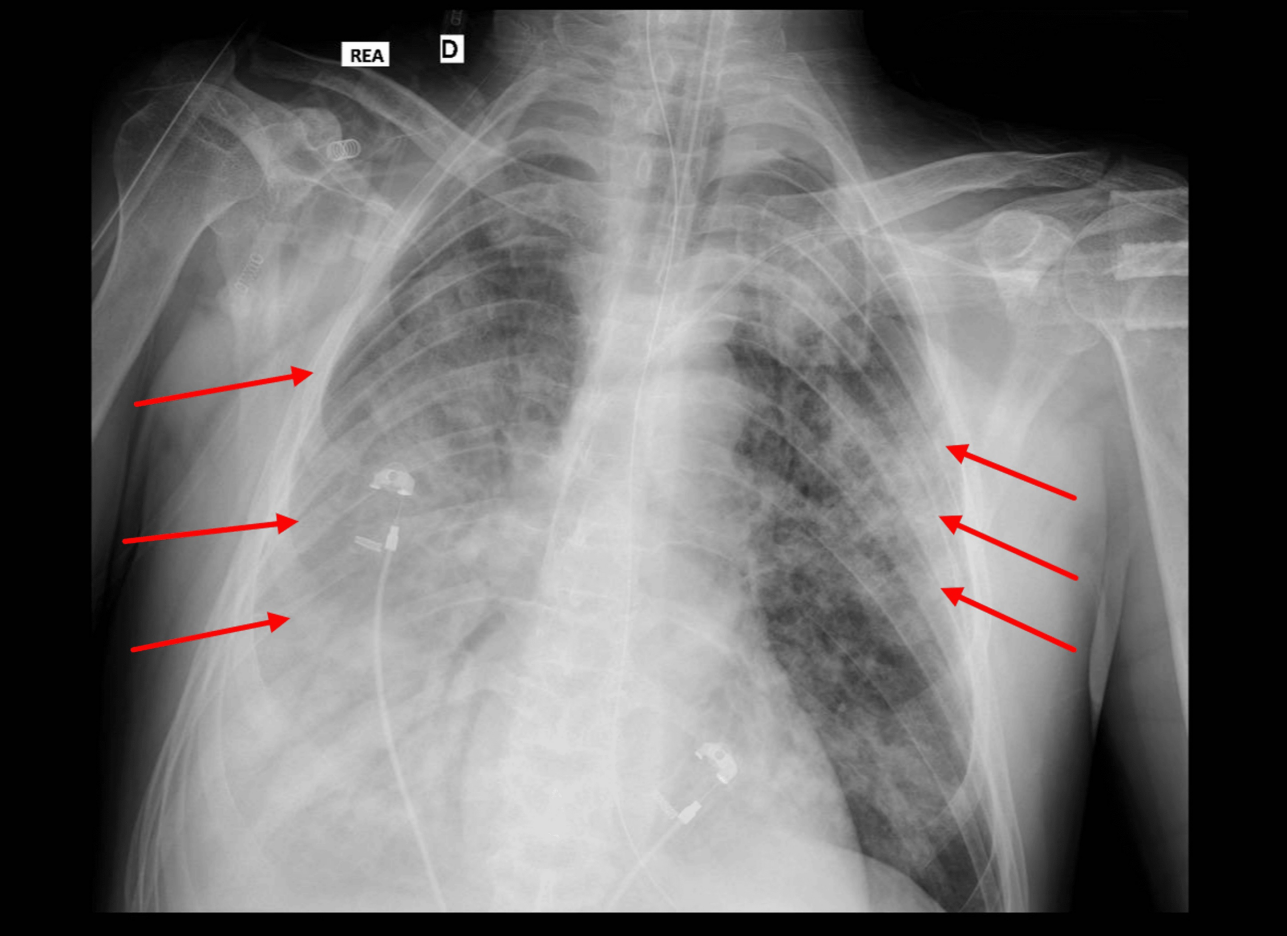
Chest X-ray showing bilateral pulmonary infiltrates with the right white lung

Despite appropriate antimicrobial therapy, the patient's condition progressed to severe ARDS with bilateral alveolar infiltrates on chest imaging (Figure 2) and a PaO₂/FiO₂ ratio of 100. Prone positioning was performed on hospital days two and three. Septic shock developed concurrently, requiring vasopressor support with norepinephrine. Culture of tracheal aspirate confirmed L. pneumophila, establishing a definitive diagnosis of Legionnaires’ disease.

**Figure 3:**
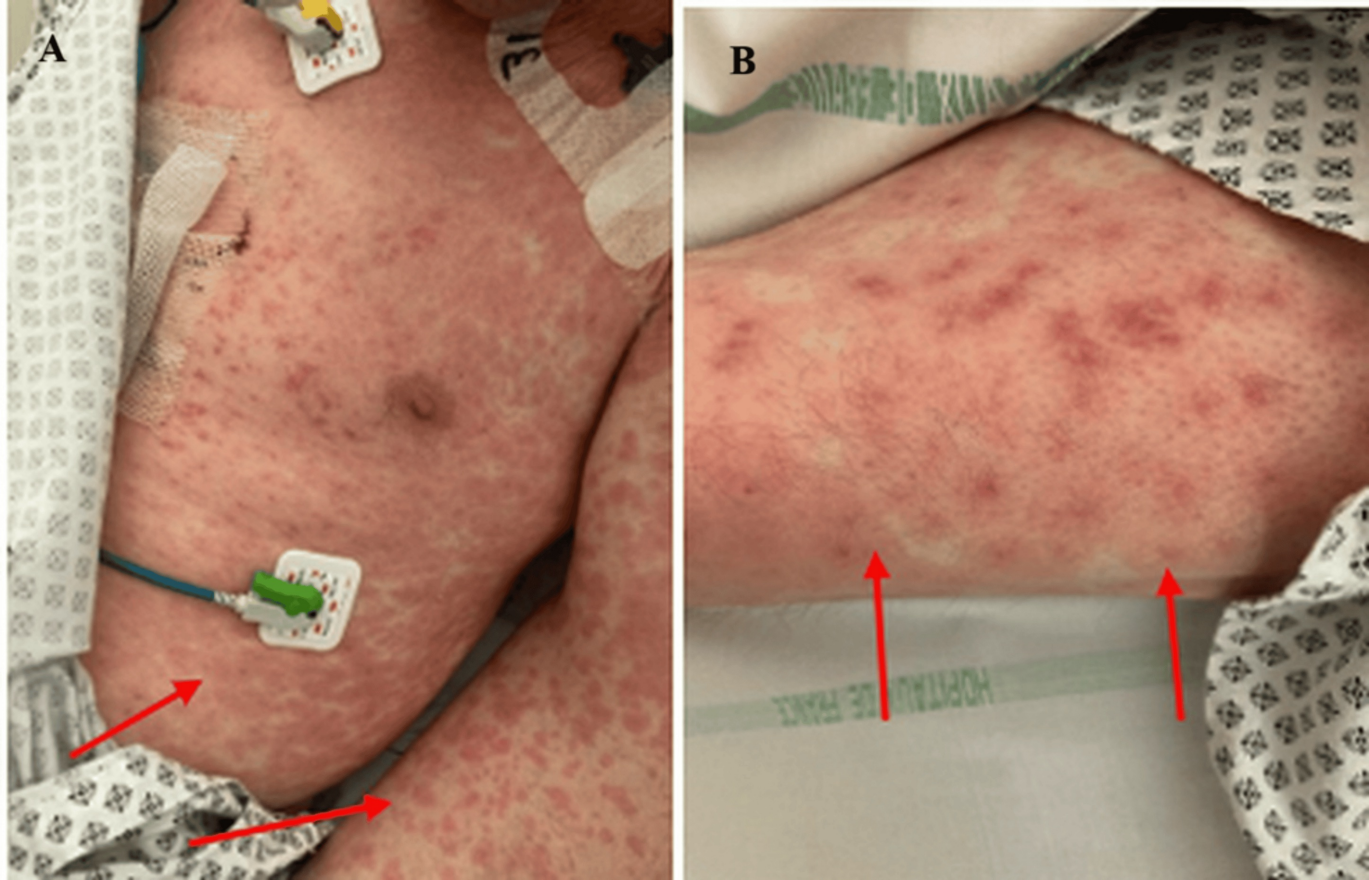
Patient's skin lesions showing macular rash with some target lesions

On day nine of hospitalization, corresponding to seven days after initiating Legionella-specific antibiotic treatment, the delayed onset of the rash suggested an immune-mediated mechanism rather than an immediate drug-related reaction, the patient developed a diffuse, non-pruritic, erythematous maculopapular rash (Figure 3). The eruption was bilateral and symmetrical, involving the trunk and limbs. Lesions on the palms appeared as papular erythema, while petechial purpura were noted on the ankles. The rash had a targetoid, “cocoon-like” appearance; there was no mucosal involvement or signs of desquamation.

Drug-induced skin manifestation was suspected; thus, a skin biopsy was done revealing mild interkeratinocytic edema and a superficial perivascular lymphocytic infiltrate, with rare keratinocyte necrosis. There was no vacuolar degeneration of the basal layer and no significant eosinophilic infiltrate. Direct immunofluorescence testing was negative, and fungal cultures were negative as well. The findings were not consistent with a drug-induced toxidermia.

Polymerase chain reaction (PCR) testing for herpes simplex virus (HSV) and Mycoplasma pneumoniae-two common infectious triggers of erythema multiforme-was negative. Given the clinical, histological, and laboratory findings we concluded that the diagnosis of erythema multiforme, secondary to systemic Legionella infection.

The cutaneous lesions progressed for two days, then started to improve and disappeared completely before discharge without specific dermatologic treatment. The patient continued to receive levofloxacin for a total of 21 days and spiramycin for five days. He was extubated on day 12, weaned off vasopressors by day 10, transferred out of the ICU by day 15, and discharged from the hospital on day 20. Acute kidney injury noted during the ICU stay resolved prior to discharge.

## Discussion

This is a rare case of dermatologic manifestation in a patient with severe Legionella pneumonia, with skin lesions appearing after proper antimicrobial therapy, raising several diagnostic possibilities. Similar to the report of Ziemer et al. [4], drug-induced eruption was considered in our case, but the absence of eosinophilia, systemic hypersensitivity signs, and characteristic histopathological features argued against this diagnosis. In addition, the delayed onset of the rash in the context of ongoing targeted therapy further supports a non-immediate, immune-mediated mechanism.

In two different reports by Calza et al. and Carter et al. [5,6], skin lesions were described as macular, maculopapular, petechial, purpuric, or targetoid lesions. These findings overlap significantly with EM, an immune-mediated hypersensitivity reaction most commonly triggered by infections, particularly herpes simplex virus and Mycoplasma pneumoniae [7,8]. The pathophysiology involves a cell-mediated immune response leading to keratinocyte injury, which may also be triggered by other infectious agents [9].

In our patient, the rash closely resembled EM both clinically and histologically. After exclusion of the two major infectious triggers (HSV and Mycoplasma pneumoniae) by PCR testing, and in the context of confirmed Legionella infection, the diagnosis of Legionella-induced erythema multiforme was considered the most plausible explanation. This diagnostic reasoning is further supported by the concordance between clinical presentation, histopathological findings, and the exclusion of alternative etiologies.

A 2022 review by Carter and colleagues analyzed 12 published cases of Legionella-associated rash [6]. Only one-third of patients were immunosuppressed, suggesting that immune status may not be a major predisposing factor for dermatologic involvement. Additionally, there appears to be no consistent temporal relationship between infection onset and rash development. Cutaneous manifestations may occur early, during treatment, or in the recovery phase. However, late-onset presentations, as seen in our patient, appear to be more frequent; the onset during the treatment phase aligns with these observations and supports the hypothesis of a delayed immunologic response.

On the other hand, cutaneous manifestations due to Legionella remain too infrequent to establish clear patterns regarding distribution. Reported cases most commonly describe involvement of the trunk, chest, upper limbs, and thighs, often sparing the palms and soles. Dušek et al. reported palm involvement without plantar lesions, whereas in our case both palms and soles were affected. In addition to exanthematous eruptions, other rare cutaneous extrapulmonary manifestations of Legionella infection include panniculitis, cellulitis, and abscess formation, particularly in disseminated disease [10,11].

Overall, Legionnaires’ disease should be recognized as a systemic infection with potentially diverse and atypical manifestations, including rare dermatologic presentations that may mimic drug reactions or other immune-mediated conditions [12,13].

## Conclusions

Legionnaires’ disease is a multi-systemic illness with a well-established respiratory and systemic presentation. Cutaneous involvement, although uncommon, can occur and may resemble EM. The recognition of these dermatologic signs is important to differentiate them from drug reactions or unrelated conditions, which may complicate management.

Clinicians should consider Legionella infection in the differential diagnosis of acute rash in patients with pneumonia, especially if respiratory symptoms, electrolyte abnormalities, or systemic inflammation are present, and should distinguish it from drug eruptions to ensure continuation of essential antibiotic therapy. Further documentation of such cases, particularly those characterizing the spectrum, timing, and histopathology of cutaneous findings, may improve our understanding of the pathophysiology and clinical manifestations of Legionnaires’ disease and help guide diagnostic and management strategies.
